# Resilience, Coping Strategies and Posttraumatic Growth in the Workplace Following COVID-19: A Narrative Review on the Positive Aspects of Trauma

**DOI:** 10.3390/ijerph18189453

**Published:** 2021-09-08

**Authors:** Georgia Libera Finstad, Gabriele Giorgi, Lucrezia Ginevra Lulli, Caterina Pandolfi, Giulia Foti, José M. León-Perez, Francisco J. Cantero-Sánchez, Nicola Mucci

**Affiliations:** 1 Department of Experimental and Clinical Medicine, University of Florence, 50139 Florence, Italy; nicola.mucci@unifi.it; 2 Business @ Health Laboratory, European University of Rome, 00163 Rome, Italy; cate.pandolfi@gmail.com (C.P.); giuliafoti.98@gmail.com (G.F.); 3 Department of Human Sciences, European University of Rome, 00163 Rome, Italy; gabriele.giorgi@unier.it; 4 School of Occupational Medicine, University of Florence, 50134 Florence, Italy; lucreziaginevra.lulli@unifi.it; 5 Department of Social Psychology, Universidad de Sevilla, 41004 Sevilla, Spain; leonperez@us.es (J.M.L.-P.); fcantero@us.es (F.J.C.-S.)

**Keywords:** COVID-19 pandemic, SARS-CoV-2, trauma, growth, psychological health, workers’ wellbeing, occupational health and safety

## Abstract

The COVID-19 pandemic represents a traumatic event that has profoundly changed working conditions with detrimental consequences for workers’ health, in particular for the healthcare population directly involved in addressing the emergency. Nevertheless, previous research has demonstrated that traumatic experiences can also lead to positive reactions, stimulating resilience and feelings of growth. The aim of this narrative review is to investigate the positive aspects associated with the COVID-19 pandemic and the possible health prevention and promotion strategies by analyzing the available scientific evidence. In particular, we focus on the constructs of resilience, coping strategies and posttraumatic growth (PTG). A literature search was performed on the PubMed, EMBASE, Scopus, Web of Science, Google Scholar and Psycinfo databases. Forty-six articles were included in the literature synthesis. Psychological resilience is a fundamental variable for reducing and preventing the negative psychological effects of the pandemic and is associated with lower levels of depression, anxiety and burnout. At the individual and organizational level, resilience plays a crucial role in enhancing wellbeing in healthcare and non-healthcare workers. Connected to resilience, adaptive coping strategies are essential for managing the emergency and work-related stress. Several positive factors influencing resilience have been highlighted in the development of PTG. At the same time, high levels of resilience and positive coping strategies can enhance personal growth. Considering the possible long-term coexistence and consequences of COVID-19, organizational interventions should aim to improve workers’ adaptive coping skills, resilience and PTG in order to promote wellbeing.

## 1. Introduction

A new type of coronavirus (SARS-CoV-2) causing coronavirus disease 2019 (COVID-19) was identified in December 2019, and on 11 March 2020, the World Health Organization [WHO] declared COVID-19 a global pandemic [1]. Science has made great strides in controlling COVID-19 infection, particularly because of vaccine development. However, predictions on future scenarios point to a possible coexistence with COVID-19 for many years, underlining the need to address the psychological aftermath of the pandemic [2,3]. Pandemics and bio-disasters have been associated with detrimental consequences for the physical and mental health of individuals, especially in the case of healthcare professionals who generally carry the greatest burden [4,5]. Data from previous experiences, as in the case of Severe Acute Respiratory Syndrome (SARS) of 2003, Middle East Respiratory Syndrome (MERS) of 2013–2016 and Ebola of 2014–2016, display an alarming picture with symptoms such as anxiety, depression, burnout and post-traumatic stress disorder (PTSD) that persist even after 1–3 years [6,7]. Likewise, there is growing evidence that COVID-19 is associated with post-traumatic symptoms and psychological disorders, especially for frontline health workers [8,9]. For example, a recent meta-analysis [4] analyzed 65 studies for a total sample of 79,437 participants and highlighted a prevalence of 34.4%, 31.8%, 40.3%, 11.4%, 27.8%, 46.1% and 37.4% for anxiety, depression, stress, post-traumatic stress syndrome, insomnia, psychological distress and burnout, respectively. Furthermore, the COVID-19 pandemic is characterized by peculiarities not found in previous disasters such as prolonged insecurity and global economic and social consequences, representing a mass traumatic event [5,10].

The post-disaster mental health literature and trauma research have actually shown that adverse effects on psychological health do not always occur and that traumatic experiences can even lead to positive emotional states and growth [11,12,13,14,15,16]. Even after experiencing terrible events, the evidence suggests that people may experience some positive aspects, such as in the case of bereavement, rape, cancer, terrorism, natural disasters and even epidemics, as in the case of the MERS outbreak [14,15,16,17]. The ability to adapt to unpleasant situations and to recover quickly from trauma has been studied through the constructs of resilience, posttraumatic growth (PTG) and coping strategies [11,12,14,18,19]. Research has not yet reached unanimous agreement on the definition and conceptualization of resilience. Generally, the construct can be defined as positive adaptation despite adverse conditions or as the ability to maintain adequate functioning despite destructive events [12,20,21,22]. Posttraumatic growth defines the positive psychological change that occurs following highly stressful and demanding life situations. The positive transformation originally involved three domains, which were then extended to five thanks to the development of the Post-traumatic Growth Inventory (PTGI): “personal strength”, “relating to others”, “appreciation of life”, “openness to new possibilities” and “spiritual change” [13,14]. The concept underlying the PTG experience concerns the disruption of the individual’s belief system, which is called the “assumptive world”. The following process of emotional regulation and sense making will then lead to growth as a result of the rebuilding attempt [14,15,16]. As for the relationship between the constructs, the research results are still inconclusive [16]. For example, according to Tedeschi and Calhoun [14] the adversity experienced is greater in the case of PTG, which involves a qualitative transformation in functioning that exceeds the ability to resist traumatic circumstances. Nevertheless, some studies show a significant relationship between resilience and PTG [17,23]. Eventually, another factor that strongly affects the psychological outcomes of disaster-exposed employees refers to coping, which can be defined as the set of adaptive or maladaptive cognitive/behavioral strategies used to deal with adverse and stressful events [11,24]. Coping strategies can be generally classified as problem-focused (e.g., trying to solve the situation, address the cause) and emotion-focused (e.g., reinterpretation, distancing) or as approach-focused (i.e., strategies aimed at dealing with stressor) and avoidance-focused (i.e., maladaptive avoidance of the situation) [19,24]. Furthermore, approach-focused coping styles such as proactive behaviors, seeking social support and facing the situation are associated with greater resilience [11]. Similarly, active strategies such as problem-focused coping and active-relational coping are significantly associated with increased PTG [25].

Hence, the literature shows that the aftermath of tragic events can have a positive impact in terms of personal growth and meaning, suggesting that resilient attitudes may be more prevalent than expected [11,12,15,18]. In this regard, Bonanno [12] challenged the grief work assumption, arguing that the most common reaction to highly traumatic events are not symptoms of distress such as PTSD and depression as much as resilience. For example, analyzing the reactions of New York residents after 11 September 2001, Bonanno and colleagues [26] found resilience, defined as the absence of PTSD, in 65.1% of the sample (N = 2752). Nevertheless, this perspective does not imply that resilient people do not experience symptoms of discomfort but rather that these do not compromise the general trajectory of functioning [12,27]. Evidence shows that traumatic events can bring positive aspects with prevalence rates for growth ranging 30–70% and 40–75% for more traumatizing professions [11,15,28]. In addition, growth also occurs in cases of vicarious or secondary trauma as for professionals who work closely with victims of adverse events (e.g., health personnel, social workers) [29]. Regarding the specific context of bio-disasters, a recent study analyzed PTG in a sample of healthcare workers (HCWs) involved in the 2015 MERS epidemic in South Korea [17]. The results showed that resilience was the only significant predictor of PTG while the interviews showed that resilience was experienced in terms of the hardiness, persistence, optimism and support sub factors.

As scholars have pointed out, evidence-based approaches are needed to protect workers’ health and promote successful adaptation in the aftermath of COVID-19. However, most of the available studies have investigated the negative outcomes of the COVID-19 pandemic, highlighting a gap in research examining whether the positive aspects are achievable and how this can be done [3,11,12,18]. In light of this, the purpose of this narrative review is to collect the available evidence on the positive and adaptive aspects in the context of the COVID-19 pandemic. In particular, our main objective is to analyze the protective factors for the mental health of workers with reference to the constructs of resilience, coping and PTG.

## 2. Materials and Methods

### 2.1. Literature Research and Data Collection

A literature search was performed on the PubMed, EMBASE, Scopus, Web of Science, Google Scholar and Psycinfo databases from 15 April 2021 up to 31 May 2021. The search strategy used a combination of free text and controlled vocabulary, including the keywords “resilience”, “coping strategies”, “posttraumatic growth”, “personal growth”, “workers”, “employees”, “covid-19”, “pandemic”. A manual research was also performed screening the bibliographic references of the most relevant selected papers. The research was based on the following PICO scheme:

- Population: Workers from any sector.
- Intervention: The consequences of the COVID-19 pandemic in the workplace.
- Comparison: Not considered.
- Outcome: Resilience, coping strategies, posttraumatic growth, personal growth.

Two independent reviewers (G.L.F. and L.G.L.) read titles and abstracts of the papers identified by the search strategy and carried out a first screening; a further selection was subsequently made by analyzing the full text of the articles. Investigators provided their judgment on the inclusion of each document separately, and disagreements were resolved by discussion with a third reviewer (C.P.). Data were manually extracted in a chart developed jointly by the authors, including title, authors, year of publication, type of study, type of job, sample, country where the investigation took place, aims and variables analyzed and a short summary of the findings. In this article, we merged title, authors and year of publication into the category “reference” (see Table 1). After the collection of the relevant data, a synthesis of the evidence was performed following a qualitative and narrative approach. In particular, the findings for each topic (posttraumatic growth, resilience, coping strategies) were collected together, identifying similarities and differences between the selected studies, as well as relationships, risk factors and outcomes.

### 2.2. Inclusion Criteria

The inclusion criteria followed the PICO scheme mentioned above; in particular, we included articles focusing on positive mental aspects related to the COVID-19 pandemic in the workplace, including articles analyzing resilience and coping strategies applied by workers to deal with the psychological strain of managing the pandemic at work. Articles analyzing posttraumatic growth and personal growth consequent to pandemic were also included.

### 2.3. Exclusion Criteria

Articles written in languages other of English were excluded. Reports of less academic significance, letters to the editors, non-peer reviewed articles, individual contributions and purely descriptive studies published in scientific conferences without any quantitative and qualitative inferences were excluded from this review. Furthermore, review articles were not included in the literature synthesis but discussed in other paragraphs. Studies regarding the general population and not specifically focused on workers were also excluded.

## 3. Results

**Figure 1:**
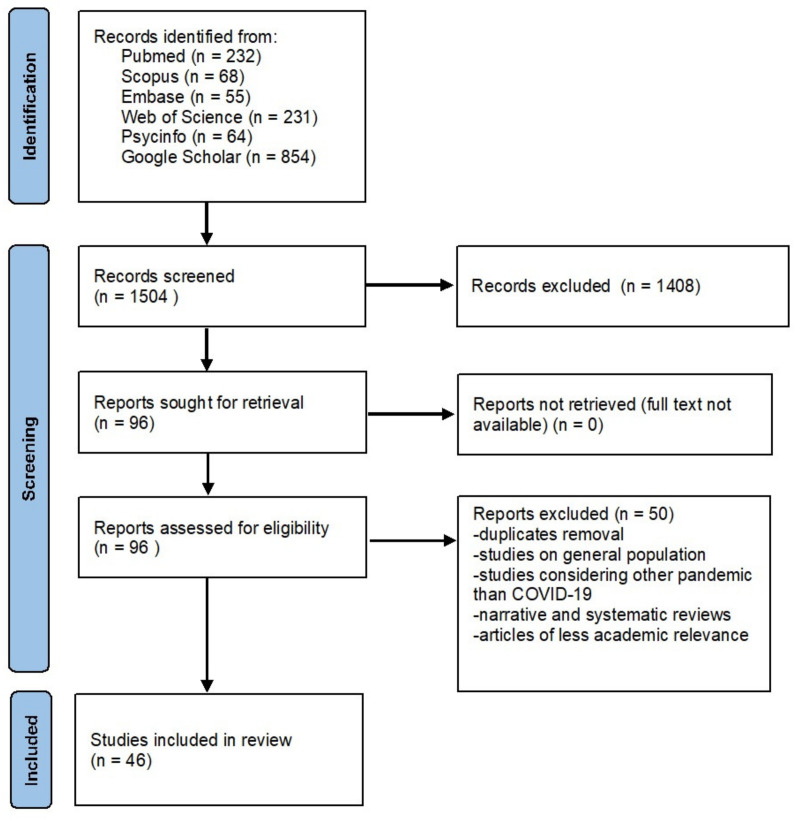
Flow diagram of the literature search and articles selection (adapted from PRISMA 2020 guidelines for systematic reviews) [76].

The online search retrieved a total of 1504 papers: Pubmed (232), Scopus (68), Embase (55), Web of Science (231), PsycInfo (64) and Google Scholar (854). Among these studies, 1444 records were excluded because they did not match the inclusion criteria, while 57 full texts were assessed for eligibility. After removing duplicates, 46 articles were included in the literature synthesis. The process of the literature search and the selection of papers is shown in Figure 1. The included studies and the main findings are included in Table 1.

The articles selected were published in several countries, representing a comprehensive sample from various parts of the world, except for Africa and South America, where no published article met the inclusion criteria. The most representative area was Europe (15 articles, 32.7% of the total) and among these, Italy was the country where most of the research was conducted (seven articles, 15% of the total). Among the individual countries, China was the most represented with 10 articles (21.3%). Six articles were published in North America (five in USA and one in Canada, 12.8%). Israel (2), India (2), Pakistan (1), Iran (1), Korea (2), Japan (1) and South-East Asia (2) were also present.

Three major topics were identified and analyzed: resilience, coping strategies and posttraumatic growth. The findings for each topic are described in the following paragraphs and further summarized in Table 2.

### 3.1. Resilience

Despite the numerous negative psychosocial effects of the COVID-19 pandemic, positive consequences in the workplace are also possible. Among the selected studies, 25 articles analyzed resilience during the COVID-19 pandemic, alone or together with other constructs. Studies concerning this aspect mainly included healthcare professionals as a sample (nurses, surgeons, medical assistants, etc.) and, to a lesser extent, non-healthcare workers. All the studies analyzed followed a cross-sectional design except for two longitudinal studies, one prospective controlled trial and one predictive study.

Resilience was associated in different ways with positive and negative (growth/stress) lockdown outcomes. Resilience correlated with Secondary Traumatic Stress (STS), age correlated with PTG, while education and nearly all coping strategies correlated with both STS and PTG [33]. In Chinese nurses working during the COVID-19 emergency, resilience had the strongest direct effect on intention to stay and significantly influenced PTG and perceived professional benefits [35]. In a study concerning moral injury among healthcare workers in the US [39], moral injury remained stable over three months, while distress decreased but was not affected by any baseline occupational or resiliency factors. Moreover, resilience played a mediating role between depression and burnout [40,62,70]. Individuals at high risk of burnout showed significantly lower levels of resilience [66]. Furthermore, an Inquiry-Based Stress Reduction (IBSR) intervention improved resilience for a sample of teachers in Israel [41]. Through resilience, emotion-focused strategies were negatively related to psychological distress directly and indirectly in a sample of Spanish nurses [42]. Resilience was negatively correlated with depression, stress and anxiety [44,46,54,65,68,71]. Age, work experience and level of education had a significant positive correlation with nurses’ resilience score [45]. Resilience had a positive and significant correlation with life satisfaction, positive affect, perceived social support, participants’ age, adoption of personal precautions against coronavirus, nutrition and sleep quality [52]. A Chinese study [53] found that resilience positively predicted PTG and vice versa, creating a cycle of reinforcement between resilience and PTG over time. In addition, burnout was negatively associated with both resilience and PTG. A study conducted on a sample of Malaysian employees showed that resilience was significantly associated with work engagement. Furthermore, self-efficacy influenced work engagement directly and indirectly through resilience [55]. A research carried out on a sample of Vietnamese tourism employees highlighted that core beliefs challenge was positively related to workers’ resilience while cognitive reappraisal played a mediating role in this relationship [59]. Resilience also plays a crucial moderating role in the relationship between PTSD and PTG: High levels of resilience enhanced PTG beyond the mean level [60]. In another study carried out on a sample of Chinese front-line nurses, resilience was negatively associated with PTSD [61]. An Indian study conceptualized a resilience “framework” pointing out three concepts: Forming a “resilient identity”, the resilience “management” and working through socio-occupational distress [63]. Canadian research [64] underlined the importance of resilience factors (i.e., trait resilience, family functioning, social support, social participation and trust in healthcare institutions) in association with mental health and well-being. Lower stress correlated with higher trait resilience, which, among the five factors, seemed to be the most important. Resilience is also a mediator between the effects of social support and mental health among HCWs [69].

### 3.2. Coping Strategies

Sixteen of the studies (34%) included in the review analyzed coping strategies applied in the workplace to deal with the pandemic emergency. Most studies used a cross-sectional design and surveys to explore the type of coping strategies and their association with psychological outcomes. Only two studies used a qualitative approach and one study employed a longitudinal design. Fourteen studies explored coping strategies among healthcare workers, while the other two investigated this topic in teachers and mechanical Turk workers, respectively. In healthcare workers, coping strategies seem to play a fundamental role in the management of the emergency and the related occupational stress. Positive and negative coping strategies were identified. The former were associated with a reduction in poor mental health outcomes [38,42,51,66,71,72,74,75]. Active coping strategies were also positively associated with resilience [71]. The key positive coping strategies were a positive attitude towards the problem, social network, peer support, teamwork, self-reliance, problem negotiation and self-care [38,63,74]. Seeking social support was a common coping strategy. In addition, in some studies, this strategy was adopted to a greater extent by workers with lower mental well-being [38,50]. A problem-focused attitude was found to be a protective factor for reducing anxiety and depression [38,48] while another study highlighted an association between this coping strategy and higher levels of nurses’ psychological distress [42]. Emotion-focused strategies were negatively related to nurses’ psychological distress directly and indirectly through resilience [42] and were mostly employed by men [48]. Comparing problem and emotion-focused coping strategies, only problem-focused coping was effective in reducing PTSD symptoms, mediating the positive effect of organizational support [75]. PTG was also linked to coping strategies, being predicted by a mixture of adaptive and maladaptive strategies [33]. Escape-avoidance coping strategy was common [50] and was associated in some studies with a higher level of stress along with overcommitment [33,38,43,49]. At the same time, as pointed out by Maiorano et al., [72] avoidance strategies allowed workers in the first phase of emergency to limit their sense of helplessness and inability, favoring resilience and the activation of proactive attitudes. Religious practices were also investigated as coping strategies, being highlighted as common [43] but not significant in reducing stress levels [38,51], although workers with higher levels in the hope/optimism dimension in the field of spirituality showed less coronavirus-related anxiety [51]. Finally, lower age and female gender, along with lower resilience and less adaptive defensive functioning, were predictors of stress [38,50,66]. Even in non-healthcare workers, coping strategies were common methods for dealing with the new conditions dictated by the pandemic [34]. In teachers, similarly to healthcare workers, avoidance coping strategies were associated with higher stress and reduced positive psychological outcomes [56].

### 3.3. Posttraumatic Growth (PTG)

Among the selected studies, nine articles (19%) analyzed PTG during the COVID-19 pandemic, alone or in combination with the other constructs. All of the selected articles were studies analyzing prevalence, level and possible association of PTG in healthcare workers who dealt with the health emergency at some level. Six articles out of nine (66%) explored the PTG of nurses, while the other three had generic healthcare workers as a sample. Most of the studies used the Post Traumatic Growth Inventory Scale (PTGI by [13]) to assess the impact of this variable on HCWs, one study used the Changes in Outlook Questionnaire (CIOQ by [77]) and one study used a qualitative approach through interviews with the subjects involved. Pandemic-related distress and growth are connected in a complex relationship that depends on intra- and inter-personal factors [60]. In nurses working in the COVID-19 emergency, an intensification of traumatic stress symptoms has emerged, for example regarding symptoms of avoidance [31]. At the same time, they also reported positive changes in the existing situation, which may be an expression of adaptation in the form of PTG [31]. In particular, healthcare workers working on the frontline seem to have higher levels of PTG compared to non-frontline healthcare workers [32,36,58]. Some factors have been recognized as being associated with PTG: In one study [30], PTG was influenced by the length of service, self-confidence in frontline work and psychological intervention or training during the epidemic. Workers have often experienced PTG through “deliberate rumination”, a process of seeking value and meaning to their own experience [30,32] or through the tendency to positively reappraise events [60]. Fear of contagion and awareness of the risk were found to be associated with PTG [30,37] while lack of personal accomplishment was a key negative influence factor [36]. In another study [33], a combination of adaptive coping strategies predicted the level of PTG, as described also by study [58]; passion for work was also a determinant for the development of PTG [37]. Other personal factors associated with PTG were sex, fertility and marital status [58]. Moreover, resilience seems to play an important role in the development of PTG, as reported in study [35], which showed that the higher the nurse’s resilience, the easier it is to perceive professional benefits, which results in stronger intent to continue working on the frontline. High levels of resilience enhanced growth beyond mean and clinically relevant levels of PTSD [60].

## 4. Discussion

Since the beginning of the COVID-19 pandemic, scholars have collected substantial evidence regarding the tremendous impact of this situation on the workforce, especially in the case of healthcare workers dealing directly with the disease [8,78,79]. The sudden and massive outbreak of COVID-19 has overwhelmed even the most advanced healthcare systems and has significantly affected almost all business sectors, leading to the need for organizational changes. After an initial pandemic phase characterized by a significant lack of resources, the situation continues to exert extreme pressures on healthcare professionals [80,81]. Furthermore, in non-health settings, the economic crisis, the implementation of safety and contagion measures, the adoption of remote work, increased and decreased workloads and the overall uncertainty about the future have negatively affected the mental health of workers in several economic sectors [78]. To the best of our knowledge, this review represents the first attempt to comprehensively analyze the positive aspects of COVID-19 seen as a traumatic experience in the workplace. Indeed, a better understanding of the mental processes underlying traumatic experiences and their determinants seems crucial in planning occupational safety and health practices.

Most of the retrieved articles considered healthcare professionals as a sample, as the literature has extensively analyzed the impact of the sanitary emergency on these workers. Despite the negative mental effects, dealing with the COVID-19 pandemic has forced workers to develop resilience strategies, as during other outbreaks [17]. As already mentioned, resilience is generally defined as the ability to adapt and maintain adequate functioning despite adverse events and can be conceptualized as a trait, outcome or process [20,21,22]. For healthcare workers, coping with mental health problems such as anxiety, depression and burnout during the emergency can be challenging. Overwhelmed by the workload, the lack of material and human resources, workers also face an increased risk of ‘moral injury’ when addressing the ethical challenges of the pandemic and the discrimination experienced due to the fear of contagion by the general population [82,83]. As evidenced by previous research, psychological resilience is a fundamental variable in reducing and preventing the negative psychological effects of the pandemic [18]. In our review, we found that resilience is associated with lower levels of depression, anxiety and burnout [44,46,53,54,65,68,71]. Resilience improves personal growth and perceived professional benefits [33,53] and has a positive impact on work engagement even in non-healthcare workers [28]. Overall, we found that age and work experience positively correlate with aspects of resilience in workers. A relevant point is that resilience is considered not only at the individual level, as a key role is played by the organizational resilience mechanisms that shape the way healthcare professionals experience the crisis [3,84,85]. Resilience seems to be a pivotal variable in dealing with work-related stress, even in the toughest situations, such as the COVID-19 pandemic. The close relationship between the organizational and personal levels underscores the need for practical measures to support and strengthen resilience, including education, resilience training and interventions to create the feeling of being prepared [86,87] Furthermore, interventions should focus on young and less experienced workers, as they are the most vulnerable in terms of developing resilience. Closely related to resilience, we found that coping strategies play a fundamental role in the management of the emergency and the related occupational stress. Negative coping mechanisms like escape and avoidance strategies or overcommitment seem to be associated with worse mental outcomes [33,38,43,49,56], while positive attitude towards the problem, social network, peer support, teamwork, self-reliance, problem negotiation and self-care [38,63,74] play a positive role in reducing stress and boosting resilience. Consistent with research on previous epidemics/pandemics, dysfunctional attachment and maladaptive coping have been highlighted as risk factors for reduced mental well-being [86]. At the same time, resilience indicators (hardiness, vigor) and self-efficacy were found to be protective factors for good mental health outcomes [88]. As pointed out from previous research [89] we found some differences between different categories of healthcare professionals, with nurses experiencing less resilience and more occupational stress [65]. In our view, this may be partly related to the tremendous workload of nursing professionals in caring for COVID-19 patients, who have demanding needs (e.g., pronation in ICU). Interestingly, being a female worker and having less work experience appear to be negative factors for developing adequate forms of coping and resilience, and this may be associated with previous findings on the need for specific training and education to build resilience mechanisms [66,72,86]. Arguably, professional experience and higher education levels can be seen as protective factors, as ‘experienced’ workers have more psychological and even professional resources to learn from the disaster rather than being overwhelmed. The negative association between the female gender and the development of effective coping strategies is consistent with previous research on this aspect. Indeed, women tend to adopt emotion-focused strategies to change their feelings, and these types of strategies can be less effective in coping with stressful situations than problem-focused methods [75], which are more common among men. However, other studies suggest that male workers [48] also adopt emotion-focused coping strategies and more research is needed to explain the mechanisms underlying gender differences. Fighting daily with the virus, as in the context of pandemics, can be considered a form of bio-disaster and traumatic experience. For those involved in the recovery and relief efforts during and after a disaster, the experience has frequently been reported as fulfilling, worthwhile, rewarding and meaningful and can make workers feel they have benefited both personally and professionally [90,91,92,93,94,95]. In the context of the COVID-19 pandemic, posttraumatic growth forms have been detected in healthcare workers after the early stages, especially in those on the front line [32,36,58]. Several positive factors influencing resilience have also been highlighted in the development of PTG, such as the length of service, self-confidence in frontline work and psychological intervention or training during the epidemic [30]. It seems that more experienced workers express a higher level of PTG, probably due to a higher initial level of awareness, as previously highlighted. At the same time, high levels of resilience and positive coping strategies enhance personal growth so that intervention fostering resilience are likely to help develop PTG [30,36,58].

### 4.1. Strengths and Limitations

This narrative review represents one of the first attempts to identify the possible positive aspects associated with the COVID-19 pandemic in the workplace. To the best of our knowledge, this is the first comprehensive review addressing personal and psychological growth during and after the COVID-19 pandemic. This review provides in-depth insight into the positive mechanisms underlying workers’ resilience, especially HCWs. Identifying such positive associations seems fundamental to guide policy makers and stakeholders towards the future organization of work. Despite its narrative approach, this review used PRISMA compliant method to search the literature, adding value to the evidence retrieved [76]. Nevertheless, despite having followed the guidelines of the literature, some limitations should be addressed. The included studies were conducted in several countries with differences in terms of culture and healthcare systems. The pressure on the workforce may vary according to the type of pandemic management, the level of material and immaterial resources and the time of the pandemic in which the study was conducted while social norms could shape the psychological response, resulting in different experiences of the traumatic event [8,96]. Hence, the level of resilience, PTG and the type and role of coping strategies could have been influenced by the different external circumstances (e.g., prolonged versus brief COVID-19 experience could influence the level of PTG as it takes longer to develop [35]). Another limitation could lie in the heterogeneity of the measurements and in the fact that most of the studies relied on self-report measures. Even if this review includes longitudinal studies that investigate PTG resilience and coping strategies (e.g., [37,39,75]), the majority of the studies employed a cross-sectional design, limiting the strength of the conclusions and the possibility of making causal inferences on the relationships between the variables. A further limitation concerns the lack of heterogeneity of occupations, with most of the research conducted in the healthcare domain with healthcare workers samples (e.g., nurses). Nevertheless, this population was the most affected by the COVID-19 pandemic, making it a suitable target for studying the consequences of traumatic experiences [4,8]. Future reviews could investigate the role of other positive psychology constructs in determining post-disaster mental health outcomes [14,21].

### 4.2. Practical Implications

This narrative review offers interesting insights into the possible positive outcomes of the COVID-19 pandemic seen as a mass traumatic event and stimulates reflection on what kind of interventions could be implemented. Indeed, health promotion and prevention strategies are essential to foster successful adaptation to challenging environmental conditions [4,5,83]. Previous research has highlighted that the constructs of resilience, PTG and coping strategies are intertwined in a complex relationship (e.g., [23,25]). For example, fostering PTG can lead to enhanced self-efficacy, cognitive flexibility, resilience and better coping skills. Similarly, greater resilience can lead to higher levels of PTG creating a virtuous cycle, as suggested by longitudinal findings [53]. Hence, organizations should implement interventions to foster resilience, PTG and adaptive coping through counselling services, social connection strategies and targeted training with the aim of creating positive cycles [11,33]. For example, trauma risk management has proven to be an effective strategy for enhancing the ability of workers to provide support to other colleagues, thus creating a growth environment, while practices such as self-care, small group discussions, mindfulness programs, computer-based trainings and competency training are effective in promoting resilience [3,11]. Some practical implications are listed in Table 2.

## 5. Conclusions

The COVID-19 pandemic can be analyzed as a traumatic event that can lead to detrimental consequences for the health of the workers, in particular for the healthcare population and for those directly involved in the management of the emergency. However, positive outcomes are also possible, as underlined by the trauma literature on resilience, coping strategies and posttraumatic growth. Considering the possible coexistence with COVID-19 and the long-term consequences, organizational interventions should be aimed at improving adaptive coping skills, resilience and the PTG of employees, thus leading to fulfilling experiences in a virtuous circle.

## Figures and Tables

**Table 1:** Summary of articles included in the narrative review.

Reference	Research Design/Sample/Nation	Variables Considered/Main Aims	Brief Summary	Topic
Cui et al. [30]	Cross-sectional/167 frontline nurses/China	Post-traumatic growth (PTG) prevalence/rumination/socio-demographic variables.	In the sample of nurses, the level of PTG was medium to high. This was influenced by years of work, self-confidence with respect to frontline work, risk awareness, psychological training and deliberate rumination.	PTG
Nowicki et al. [31]	Cross-sectional/325 nurses/Poland	Level of post-traumatic stress/perceived social support/opinions about the positive and negative consequences of the pandemic/sense of security and sense of meaning.	The team of nurses presented with intense post-traumatic symptoms with high avoidance symptoms. The pandemic had induced a reduced sense of security with an intense reflection on issues related to the security of oneself, others and the world. The respondents valued the meaning of life but were less likely to seek it out. In addition, PTG was detected, highlighted by positive changes in the existing situation.	PTG
Lee and Lee [32]	Qualitative study/Nurses with at least 1 year of general experience and 2 months of experience in a COVID-19 ward/South Korea	Experience in patient care in a COVID-19 hospital.	Nine themes were identified: pushed onto the battlefield without any preparation, fighting on the front line, altered daily life, low morale, unexpectedly long war, ambivalence towards patients, forces that keep me going, giving meaning to my work and taking another step forward in one’s own growth. Nurses who have interfaced with COVID-19 patients have experienced both negative and positive consequences such as PTG.	PTG
Kalaitzaki and Rovithis [33]	Cross-sectional/673 healthcare workers/Greece	The role of resilience and coping strategies in the secondary stress of Greek health workers and PTG following lockdown. Secondary traumatic stress (STS) and vicarious posttraumatic growth (VPTG).	The results suggested that greater resilience corresponded to lower levels of STS and higher levels of VPTG. PTG was associated with the use of positive coping strategies.	PTG, RESILIENCE AND COPING STRATEGIES
Kowalski et al. [34]	Brief report/179 Mechanical Turk workers/United States of America (USA)	Benefit seeking, PTG, coping style (positive reframing), optimism/pessimism, gratitude, general well-being, personal satisfaction.	Results showed a decline in satisfaction with work, leisure, fitness, mental health and finances in the midst of the pandemic. Benefit finding in relation to COVID-19 was significantly related to PTG, coping, gratitude and mental health. The most common positive aspects of the pandemic included more time with family and friends, a slower pace of life and improvements in physical health.	PTG AND COPING STRATEGIES
Liu et al. [35]	Cross-sectional/200 nurses/China	PTG, perceived job benefits, resilience and intention to stay at work.	The results showed that resilience had the strongest direct effect on intention to stay. Perceived professional benefits were a mediating factor in the association between resilience and intention to stay. The multiple serial mediations of PTG and perceived occupational benefits in the relationship between resilience and intention to stay were statistically significant.	PTG AND RESILIENCE
Chen et al. [36]	Cross-sectional/12,596 nurses/Not available (N.A.)	Assessment of trauma, burnout, PTG and influence of associated factors such as age, gender, level of education, assignment, affiliated department and patient care with COVID-19.	Nurses, ICU nurses, nurses working in COVID-19 hospitals and wards involved in treating COVID-19 patients all have worse mental health outcomes.	PTG
Moreno-Jiménez et al. [37]	Longitudinal study/172 health professionals/Spain	Workload, fear of infection, lack of staff and personal protective equipment (PPE), harmonious passion, STS and PTG.	Results revealed that in the second survey, workload and fear of contagion were positive predictors for STS, whereas harmonious passion was a negative predictor. Fear of contagion at time 1 and time 2 seemed to positively predict PTG, as did harmonious passion. Lack of staff/PPE, appeared to be a moderator as PTG was greater when workload was high, particularly in those with a high staff/PPE shortages.	PTG
Babore et al. [38]	Cross-sectional/595 healthcare professionals/Italy	Protective and risk factors for stress with reference to: socio-demographic variables, direct exposure to COVID-19 and coping strategies	The results suggested that a positive attitude towards the stressful situation was the main protective factor. Social support seeking, avoidance strategies and working with COVID-19 patients were risk factors.	COPING STRATEGIES
Hines et al. [39]	Longitudinal study/96 healthcare workers/USA	Moral injury and distress scores are expected to be higher during the first three months of the COVID-19 pandemic and should be affected by resilience factors such as job type, social support and sleep problems.	Moral injury scores remained stable for three months, while distress decreased. A favourable work environment is associated with lower moral impairment, whereas a stressful and less favourable environment is associated with increased moral impairment. Distress was not influenced by any occupational or resilience factors at baseline, except for poor sleep.	RESILIENCE
Serrão et al. [40]	Cross-sectional/2008 healthcare workers/Portugal	Analysis of the mediating role of resilience in the relationship between depression and burnout (personal, work-related and patient-related).	Psychological resilience partly mediated the relationship between depression and all three dimensions of burnout.	RESILIENCE
Zadok-Gurman et al. [41]	Prospective controlled trial with an intervention group (N = 35) and a comparison control group (N = 32)/67 teachers/Israel	Evaluation of the effect of IBSR on resilience, burnout, mindfulness and stress among teachers during the COVID-19 pandemic.	Mixed IBSR intervention improved teacher resilience and psychological and subjective well-being. At the same time, the control group experienced higher levels of burnout and a reduction in well-being.	RESILIENCE
Lorente et al. [42]	Cross-sectional/421 nurses/Spain	Evaluation of the impact of stressors (work overload, insufficient preparation to cope with job demands, lack of support, death and fear of infection) during the peak of the COVID-19 pandemic on the psychological distress of nurses. In addition, the mediating role of problem-focused and emotion-focused coping strategies and resilience is analysed.	All stressors have a significant, direct and negative relationship with nurses’ psychological distress; emotion-focused strategies are negatively related to nurses’ psychological distress directly and indirectly through resilience; problem-focused strategies are positively related to nurses’ psychological distress and negatively and indirectly through emotion-focused strategies.	RESILIENCE AND COPING STRATEGIES
Munawar and Choudhry [43]	Qualitative study/15 frontline emergency HWCs/Pakistan	Coping strategies and COVID-19 psychological impact.	The most frequently used coping strategies were religious coping and a passion to serve humanity and the country.	COPING STRATEGIES
Hong et al. [44]	Cross-sectional/824 nurses/South Korea	The effect of resilience on mental health, work-related stress and anxiety in relation to the COVID-19 pandemic.	A high level of general anxiety, work-related stress during viral epidemics and a low level of resilience were predictors of depression in healthcare workers.	RESILIENCE
Afshari et al. [45]	Cross-sectional/387 nurses/Iran	Resilience score and demographic predictive factors among nurses working in hospitals involved in addressing COVID-19.	The results of this study showed that age, work experience and education level had a significant positive correlation with nurses’ resilience score. The nurses’ resilience score was low. Multiple regression analysis indicated that work experience and level of education were predictors of resilience.	RESILIENCE
Mosheva et al. [46]	Cross-sectional/1106 doctors/Israel	Association between pandemic-related stressors (PRSF) and anxiety and assessment of the potential effect of resilience on anxiety.	The results show a negative association between resilience and anxiety. Four salient PRSFs (mental exhaustion, anxiety about being infected, anxiety about infecting family members and sleep difficulties) were positively associated with anxiety scores.	RESILIENCE
Shechter et al. [47]	Cross-sectional/657 healthcare professionals/USA	Distress, coping and preferences for support	61% of participants reported a greater sense of meaning/purpose from the COVID-19 outbreak. Physical activity/exercise was the most common coping strategy (59%) and access to an individual therapist with self-guided online counselling (33%) was of greatest interest.	COPING STRATEGIES
Huang et al. [48]	Comparative study/802 in total: 374 nurses and 430 nursing students/China	Comparison of emotional responses and coping strategies between nurses and students.	For the group of professional nurses, anxiety, fear, sadness and anger were significantly higher than for college students. More nurses used problem-focused coping strategies than students did.	COPING STRATEGIES
Vagni et al. [49]	Cross-sectional/210 in total: 121 healthcare workers and 89 emergency workers/Italy	Investigation of the relationship between coping strategies used by healthcare professionals and emergency workers to manage emergency-related stressors COVID-19.	The results suggested that the healthcare worker group had higher levels of stress and arousal and used more problem-focused coping strategies than the emergency worker group.	COPING STRATEGIES
Tahara et al. [50]	Cross-sectional/661 healthcare workers/Japan	Analysis of risk and coping factors associated with the mental state of healthcare workers.	The results suggest that female gender, low levels of communication with friends and high anxiety were associated with poorer mental health. Conversely, good health, high job satisfaction and high satisfaction with new activities were associated with a reduction in mental health problems. Most participants chose an avoidance strategy, and participants with poorer mental health were more likely to adopt social support seeking as their coping strategy.	COPING STRATEGIES
Prazeres et al. [51]	Cross-sectional/222 healthcare workers/Portugal	The role of religious-spiritual coping in relation to COVID-19 fear and anxiety in health care workers.	Participants with higher levels on the hope/optimism dimension of the spirituality scale showed less anxiety related to COVID-19.	COPING STRATEGIES
Bozdag and Ergu [52]	Cross-sectional/214 healthcare workers/Turkey	Assessment of the psychological resilience of healthcare workers taking into account individual and environmental variables.	Psychological resilience was significantly and positively correlated with life satisfaction, positive affect, perceived social support subscales, age, adoption of personal COVID-19 precautions, diet and quality of sleep while was negatively correlated with negative affect, feeling personally at risk as a healthcare professional and worried about being infected.	RESILIENCE
Lyu et al. [53]	longitudinal study 1—cross-sectional study 2/134 frontline healthcare workers in the first phase and 401 frontline healthcare workers in the second phase/China	The study explores the longitudinal relationship between resilience, PTG and the role of burnout.	Cross-lagged analysis showed that resilience at Time 1 positively predicted PTG at Time 2, which in turn positively predicted resilience at Time 3. PTG at Time 1 also positively predicted resilience at Time 2 (Study 1). Job burnout was negatively related to both resilience and PTG; in particular, emotional exhaustion moderated the link between PTG and resilience (Study 2).	PTG AND RESILIENCE
Huffman et al. [54]	Cross-sectional/785 healthcare providers, medical trainees and administrators/USA	The impact of the COVID-19 pandemic on the psychological well-being of healthcare workers, medical trainees and administrators and identify sources of stress.	Greater resilience was associated with less stress, anxiety, fatigue and sleep problems. Additionally, increased resilience and grit were protective factors in managing personal and systemic stressors at the height of the COVID-19 pandemic.	RESILIENCE
Oluwaseyi Ojo et al. [55]	Cross-sectional/259 employees/Malaysia	Based on the conservation of resources theory, the study investigates the work, social and personal resources underlying employee resilience and the impact of resilience in stimulating work engagement during the COVID-19 pandemic.	The results of the path modelling analysis showed a significant impact of self-efficacy, facilitating conditions and support from family and friends on employee resilience. Furthermore, resilience was significantly associated with engagement.	RESILIENCE
MacIntyre et al. [56]	Cross-sectional/600 language teachers/Several countries from Europe, North America, South America, Asia, Middle East	The survey measured stressors and 14 coping strategies grouped into two types, ‘approach’ and ‘avoidant’.	Correlations suggested that positive psychological outcomes (well-being, health, happiness, resilience and growth during trauma) were positively correlated with coping and negatively correlated with avoidance. Avoidant coping increased as stress increased, demonstrating a possible cost of using avoidant coping strategies.	COPING STRATEGIES
Vagni et al. [57]	Cross-sectional/236 in total: 140 healthcare workers and 96 emergency workers/Italy	The aim was to assess which stressors caused secondary trauma and to assess the protective role of the hardiness construct.	Healthcare workers had higher levels of stress and arousal than the emergency worker group, and those involved in COVID-19 treatment were exposed to a high degree of stress and were at high risk of developing secondary trauma. Commitment was associated with high levels of stress, arousal and intrusion, while control showed a protective function. Stress and hardiness accounted for 37% and 17% of the variance in arousal and intrusion, respectively.	COPING STRATEGIES
Li et al. [58]	Comparative study/455 nurses and 424 general population/China	Evaluation of PTG of Chinese nurses and the general population during the COVID-19 pandemic.	The results highlighted some differences in the PTGI score between nurses and the GP, both in the total score and in the 3 dimensions of new possibilities, personal strength and spiritual change. There were also differences between first-line nurses (FLN) and non-first-line nurses (nFLN). Psychological counselling from the WeChat network and self-relaxation were valid coping strategies for relieving nurses’ stress.	PTG
Tuan [59]	Cross-sectional/672 employees in the tourism sector/Vietnam	Study of the role of core beliefs challenge in promoting workers’ resilience.	The results revealed a positive association between workers’ core beliefs challenge and their resilience. Cognitive reappraisal was found to be a mediator in the relationship between core beliefs challenge and resilience, whereas no evidence was found regarding the mediating role of expressive suppression. Family strain negatively moderated the relationship between core beliefs challenge and both emotion regulation strategies.	RESILIENCE
Fino et al. [60].	Cross-sectional/202 healthcare workers/Italy	Analysis of the relationship between post-traumatic stress and PTG, the moderating role of resilience, emotion regulation and social support. Hypothesis: A high level of distress would be associated with PTG in health workers with high resilience, high emotion regulation skills and high social support.	The moderating role of resilience was significant, as indicated by the model and the PTSD-by-resilience interaction. Greater positive reappraisal of events was associated with high levels of post-traumatic growth at normal and above normal levels, but not with low levels of PTSD.	PTG AND RESILIENCE
Li et al. [61]	Predictive study/356 front-line nurses/China	Assessment of psychological well-being and factors associated with PTSD before and after nurses worked in COVID-19 wards.	The level of stress and the prevalence of PTSD increased significantly after working in COVID-19 units. Work experience of less than 2 years was significantly associated with a high risk of developing PTSD. Nurses working in COVID-19 wards were significantly more likely to suffer from PTSD than those working in other COVID-19-related units. Resilience was negatively associated with PTSD.	RESILIENCE
Zhang et al. [62]	Cross-sectional/180 front-line nurses/China	Analysis of the mediating roles of positive and negative affect in the relationship between resilience and burnout in Wuhan hospital nurses at the peak of the COVID-19 pandemic.	Resilience showed significant negative correlations with burnout and emotional exhaustion, depersonalization, reduced personal accomplishment. Resilience correlates positively with positive affect. The excellent preparation to resilience of frontline nurses and the associated positive effect can reduce the risk of burnout.	RESILIENCE
Debanjan et al. [63]	Cross-sectional/172 Physicians working at COVID-19 Hospitals/India	Understanding adversity and defining the resilience framework of doctors in COVID-19 hospitals, through a qualitative approach.	The resilience “framework” of these workers is a process that has emerged as doctors have faced fears of infection, uncertainty, stigma, guilt and social isolation. Unmet needs were flexible work policies, administrative measures for better medical protection, media sensitivity to the image of HCWs, effective risk communication for their health and social inclusion. Resilience consisted of three facets: forming a “resilient identity”, managing resilience and working through socio-occupational distress.	RESILIENCE AND COPING STRATEGIES
Coulombe et al. [64]	Cross-sectional/1122 workers from different sectors/Canada	The study aims to explore associations of potential resilience factors at multiple ecological levels (i.e., trait resilience, family functioning, social support from friends, social participation and trust in health care institutions) with mental health and well-being outcomes and their role as moderators against the negative effects of the pandemic.	Meaning of life was positively associated with trait resilience, better family functioning, greater social support from friends, social participation and trust in healthcare institutions. Lower stress was associated with better family functioning, trust in healthcare institutions and greater trait resilience.	RESILIENCE
Croghan et al. [65]	Cross-sectional/302 healthcare workers/USA	The purpose of the study was to assess the level of stress, resilience and ability to cope among HCWs during the initial stages of the pandemic and to determine inter-professional differences.	HCWs reported moderate-high stress scores, and normal levels of resilience and coping, the MD/NP/PA group had the highest resilience, while nurses had the lowest. Nurses also had higher stress levels compared to the MD/PA/NP group; older age was associated with higher resilience.	RESILIENCE
Di Giuseppe et al. [66]	Cross-sectional/233 healthcare workers/Italy	This study aimed to identify protective factors against perceived stress and burnout and factors that can improve resilience among health workers.	Mature defences were positively associated with resilience and personal accomplishment, while they have negative influences on stress and burnout. Neurotic and immature defences followed the opposite trend. Lower age, female gender, higher exposure to COVID-19, lower resilience and less adaptive defensive functioning were the best predictors of perceived stress and emotional exhaustion among healthcare professionals.	COPING STRATEGIES
Di Trani et al. [67]	Cross-sectional/267 healthcare workers/Italy	The general aim of this study was to explore the burnout dimensions among Italian HCWs during the COVID-19 emergency and to evaluate their relationships with some psychological features (resilience and intolerance to uncertainty).	HCWs at high risk of burnout showed significantly lower levels of resilience and higher levels of uncertainty intolerance. Levels of resilience and the ability to tolerate uncertainty were significant factors in determining the impact of the COVID-19 emergency on HCWs. The use of emotional strategies that allow individuals to remain in a critical situation without the need to control it seems to protect against burnout in these circumstances.	RESILIENCE
Fleuren et al. [68]	Cross-sectional/1126 healthcare workers/The Netherlands	The study aims to investigate the relationships between resilience, team social climate and depressive complaints, specifically focusing on infection-related fears as a relevant explanatory mechanism.	This model shows that personal resilience is negatively associated with depressive complaints and concerns about infections, which in turn are positively related to depressive symptoms. Team social climate is associated with a lower effect of worries about being infected and infecting others on depressive complaints. Resilience can be an important individual resource in preventing depressive complaints.	RESILIENCE
Hou et al. [69]	Cross-sectional/1472 healthcare workers/China	The study examined the effect of social support on the mental health of healthcare workers and its underlying mechanisms regarding the mediating role of resilience and the moderating role of age during the epidemic.	The results highlighted the protective role of social support in mental health among healthcare professionals. Resilience could be one of the pathways through which social support contributes to mental health. The effect of social support on mental health through resilience is attenuated in middle-aged healthcare workers compared to younger ones.	RESILIENCE
Sinu et al. [70]	Cross-sectional/120 frontline healthcare workers/India	This study aims to determine burnout and resilience levels and associated factors among frontline nurses caring for COVID-19 patients.	Resilience showed a significant negative relationship with emotional exhaustion and reduced professional efficacy. Emotional exhaustion and reduced personal accomplishments are significantly negatively correlated with total resilience, but there is no significant relationship between depersonalization and resilience. Increasing resilience among nurses will help mitigate the symptoms of burnout.	RESILIENCE
Lin et al. [71]	Cross-sectional/114 healthcare workers/China	Investigation of the resilience of non-local healthcare workers sent to support local healthcare workers in treating the COVID-19 outbreak.	Resilience correlated negatively with anxiety and depression but positively with active coping styles. Active coping, depression, anxiety and training/support provided by the respondent’s permanent hospital were significantly associated with resilience.	RESILIENCE AND COPING STRATEGIES
Maiorano et al. [72]	Cross-sectional/140 healthcare workers and 100 emergency workers/Italy	The study aims to investigate the direct and mediated effects of coping strategies and hardiness on secondary trauma among Italian medical staff and emergency workers involved in the first phase of the COVID-19 pandemic.	Physicians, nurses and rescuers were exposed to similar levels of organizational, cognitive, social and emotional stress. The use of cognitive and emotional avoidance strategies, especially in an initial emergency phase seems to allow these workers to limit their sense of helplessness and inability, favoring resilience and the activation of proactive attitudes. Stopping negative emotions seems to reduce stress levels and arousal and intrusive aspects of the trauma. Older workers showed a greater tendency to adopt avoidance strategies toward negative thoughts and emotions, but exhibited higher levels of arousal. Age seems to allow health and emergency workers to stop the intrusive aspects of the trauma, thus making them more committed to the intervention and less influenced by the consequences.	COPING STRATEGIES
McFadden et al. [73]	Cross-sectional/3425 healthcare workers and social workers/United Kingdom (UK)	This study examines the relationship between coping strategies and wellbeing and quality of working life in nurses, midwives, allied health professionals, social care workers and social workers who worked in health and social care in the UK during its first wave of COVID-19.	Positive coping strategies, such as active coping and seeking help, have been associated with higher well-being and a better quality of working life. Negative coping strategies showed an opposite trend. The most frequently used coping strategy was acceptance. Avoidance coping was associated with lower well-being. Among the more adaptive coping strategies, active coping and seeking help were found to help be protective factors. Active coping, help seeking, religion, humour, work-family segmentation, working to improve skills, recreation and relaxation and exercise were all coping strategies employed by workers (personal job resources) that protected them from low wellbeing.	COPING STRATEGIES
Roca et al. [74]	Qualitative study/22 nursing students/Spain	The study aimed to qualitatively explore the experiences and emotional responses of senior nursing students who volunteered to provide health assistance at the peak of the COVID-19 pandemic and to identify coping measures taken to deal with the situation.	The coping strategies used in the work context were teamwork, psychological assistance by the healthcare institution, seeking information on COVID-19 care and peer support, primarily through social networks. Other personal strategies included receiving support from family and friends, recreational activities, self-reliance, humour and religion.	COPING STRATEGIES
Zhou et al. [75]	Longitudinal study/107 healthcare workers/China	The study aims to examine the effects of perceived organizational support, self-efficacy and two types of coping strategies on the PTSD symptoms of frontline healthcare workers fighting against COVID-19 in Wuhan.	Perceived organizational support reduced PTSD symptoms through the mediating effect of problem-focused coping and the sequential mediating effect of self-efficacy and problem-focused coping strategies. Emotion-focused coping is less likely to be used and is not effective for reducing PTSD symptoms. Self-efficacy predicted reduction of PTSD symptoms by the mediating effect of problem-focused coping.	COPING STRATEGIES

**Table 2:** Main findings and implications analyzed by topic.

Topic	Overall Findings	Implications
Resilience	It is positively associated with PTG, life and professional satisfaction. It is negatively associated with work-related stress, anxiety and depression. Age, work experience and level of education positively correlate with resilience. Resilience boosts work engagement and plays a mediating role between depression and burnout.	Multi-level interventions are necessary to ensure the resilience of the workforce. Team leaders should build a sense of collective efficacy through effective communication and transparency. Counselling sessions, cognitive behavioral techniques and relaxation strategies are useful interventions to translate workers’ resources into workplace resilience.
Coping Strategies	Positive attitude towards the problem, peer support, self-reliance, problem negotiation and self-care are key positive coping strategies. Emotion and problem-focused attitudes are two common coping strategies associated with positive and negative mental outcomes. Escape-avoidance coping strategies are associated with higher levels of occupational stress. Younger age and female gender predict less adaptive functioning.	A supportive work environment is necessary for the development of adequate coping strategies. Organizations should devise interventions specifically designed to boost positive and active attitudes and decrease avoidance behaviors. In order to deal with the traumatic contents, workers should learn to recognize and be aware of their thoughts and emotions.
Posttraumatic growth	Frontline healthcare workers seem to have higher levels of PTG. PTG is influenced by the length of service, self-confidence, awareness of the risk of contagion and psychological intervention or training. PTG is often experienced through “deliberate rumination” or through a positive reappraisal of events. Adaptive coping strategies and resilience contribute to the development of PTG.	Organizations should guide workers in perceiving moments of extreme crisis as opportunities. Workers should be involved in specific programs and workshops dedicated to positive reinterpretation and reframing. Deliberate cognitive processing along with adequate emotional and instrumental support are among the key factors for successful growth.

## Data Availability

Data sharing is not applicable to this article, as the study did not report any new data.
